# The Association of Sports Participation With Depressive Symptoms and Anxiety Disorder in Adolescents

**DOI:** 10.3389/fpubh.2022.860994

**Published:** 2022-06-03

**Authors:** Guijun Chi, Lei Wang

**Affiliations:** ^1^China Volleyball College, Beijing Sport University, Beijing, China; ^2^Department of Physical Education, Tangshan Normal University, Tangshan, China; ^3^School of Physical Education and Sport Training, Shanghai University of Sport, Shanghai, China

**Keywords:** depression, anxiety, adolescents, sport participation, sport team

## Abstract

**Aim:**

An increasing body of evidence has evidenced that physical activity is negatively associated with depression and anxiety in adolescents, although the associations between various modalities of PA with depression and anxiety have yet to be effectively explored. This study aimed to investigate the associations between sports participation and depression and anxiety among a sample of Chinese adolescents.

**Methods:**

2,374 adolescents from eight schools were invited to participate in this survey, of whom 1,714 adolescents from grades 7, 8, 10 and 11 were included for final analysis. Depressive symptoms and anxiety disorder were assessed using the Patient Health Questionnaire−9 (PHQ-9), as well as the Generalized Anxiety Disorder−7 (GAD-7), respectively. Sport participation was assessed using a single question. Additionally, sociodemographic factors were assessed using a self-reported questionnaire. Ordinal logistic regression was used to estimate sport participation's associations with depressive symptoms and anxiety disorders separately, having controlled for all sociodemographic covariates.

**Results:**

Among all study participants, those with less participation in sport-related activities had a greater likelihood of reporting depressive symptoms (Odd ratio [OR] for never = 2.07; OR for 1–3 times per month = 1.77; OR for 1–2 times per week = 1.77), as well as anxiety disorders (OR for never = 1.61; OR for 1–3 times per month = 1.69; OR for 1–2 times per week = 1.38).

**Conclusion:**

Encouraging adolescents to engage in more sports participation would provide an effective and feasible approach for mental health promotion. Despite this study having found promising evidence, the research findings should be replicated using more improved research with an enhanced study design. Future researchers are encouraged to design and implement sports participation interventions aimed at promoting mental health among adolescents, while future China-based studies are encouraged to replicate or negate our study findings.

## Introduction

Physical activity (PA) is defined as any bodily movement that increases energy expenditure (1–3). Substantial evidence has indicated that regular PA participation is linked with numerous health benefits, for example healthy weight, improved cognitive function, in addition to enhanced cardiorespiratory fitness (4, 5). Across the literature, the associations between PA and mental health or disorder outcomes have been investigated and effectively established (6), suggesting that PA results in improved mental health status or may hinder the development of mental disorder across a spectrum of the population. In Biddle et al.'s (7) review, the authors summarized the evidence regarding the associations between PA and mental health or disorder outcomes among adolescents, while also recommending that researchers should unravel the association between various types of PA and health outcomes. Such evidence may assist with designing and implementing efficient and specific interventions aimed at promoting mental health in adolescents, because mental health problems have been a priority internationally (7). In this regard, recent studies have focused on specific modes of PA and their association with mental health or disorder outcomes (8, 9).

As an umbrella concept, an array of PA modes exist in the real world. For adolescents, sports participation is deemed a notable opportunity to promote healthy behaviors and overall development among children and youth, while also providing a way of increasing PA levels. It may be straightforwardly achieved in various settings (10), including school- or community-based contexts (11). An expanding body of evidence has suggested that sports participation is linked to numerous health benefits (12), among which mental health or disorder outcomes have been strongly focused on by researchers. Certain previous studies have investigated the relationship between sports participation and mental disorder outcomes among adolescents (13). For example, a recent systematic review identified the negative association of a higher frequency of sports participation with depressive symptoms and anxiety disorders among adolescents (14).

Despite evidence having convincingly demonstrated the significance of sports participation for preventing risks of depressive symptoms and anxiety disorders among adolescents (15), to date, no study has yet used a China-based data set, In turn, this has made researchers uncertain about the association between sports participation and mental disorders among Chinese adolescents. China is undergoing a period of rapid development, economic growth and societal transformation (16), thus making adolescents more exposed to health risks. Mental health problems are highly prevalent among Chinese adolescents, thus exerting health burdens on the family, community and entire society (17). In this regard, it is urgently necessary to identify effective approaches for preventing mental health problems. From the perspective of PA promotion, increasing PA levels would provide a feasible approach. Nevertheless, this assumption lacks China-based evidence. Moreover, little knowledge exists concerning the role of sports participation in relation to commonly occurring mental disorder outcomes among Chinese adolescents, for example depressive symptoms and anxiety disorder. Sports participation may be straightforwardly organized in different settings in China (18), including schools, as well as community and commercial sports organizations, which may provide an effective means of combatting increasingly pervasive mental health problems among Chinese adolescents. This calls for evidence-based findings to guide the action plans if possible. Nevertheless, such China-based data or evidence is extremely rare, meaning that it must be accumulated through relevant studies. This evidence accumulation can assist policymakers, researchers and practitioners to implement feasible and relevant mental health promotion actions.

To assist with resolving the literature gaps, this study aimed to analyse the associations of sports participation with depressive symptoms and anxiety disorders among a sample of Chinese adolescents.

## Methods

### Study Design and Participants

This cross-sectional study was conducted between March and October 2021 in various cities in South-eastern China. Adopting a convenience sampling method, school-aged adolescents at public middle and high schools were invited to participate in a paper-based survey. Five middle schools and three high schools agreed to join this survey. For each participating school, adolescents from grades 7–8 in middle school and grades 10–11 in high schools were targeted as study participants, because adolescents in the 9th and 12th grades were immersed in academic activities relating to the college entrance examination. These were excluded from our study due to time allocation considerations. All students in one to three classes that had been randomly selected per participating school were included (this selection was conducted by contacting the teacher). In total, 2,374 participants consented to complete the questionnaire (with permission from their parents or guardians), with the assistance of the participating schools' teachers and principals. Data were collected and analyzed anonymously. All invited participating adolescents were informed of the research aim and provided with instructions prior to signing their consent form. Participants received detailed instructions explaining how to answer the survey. For this study, after removing cases with incomplete questionnaires and abnormal answers or completion time, 1,714 participants who had provided valid information relating to the variables were included for the final data analysis. The study was approved by the university ethics committee (102772021RT071). All participants agreed to participate in the study with parents giving their written, informed consent.

### Measures

#### Sport Participation [Independent Variable]

Sport participation was analyzed using a single item, which stated: ‘over the past 12 months, did you participate in a sports team, sports club or sport-related activity?' The possible responses to this question were: never; 1–3 times per month; 1–2 times per week, as well as 3 times or more per week. This measurement question has been confirmed as a reliable and valid item for assessing young people's sports participation (10).

#### Depressive Symptoms and Anxiety [Dependent Variables]

Depressive symptoms were analyzed using the Chinese version of the 9-item Patient Health Questionnaire (PHQ-9). This instrument consists of nine items concerning experiences of depressive symptoms within the last 2 weeks. Each item is rated on a four-point Likert scale, ranging from 0 (not at all) to 3 (nearly every day). Total scores range between 0 and 27, with higher scores indicating more severe depressive symptoms. Depressive symptoms' severity may be classified based on PHQ-9 scores: 0–4 (minimal), 5–9 (mild), 10–14 (moderate), 15–19 (moderately severe), as well as 20–27 (severe) (19). PHQ-9's psychometric properties have been tested with Chinese children and provide adequate reliability and validity (20).

Anxiety disorders were assessed using the 7-item Generalized Anxiety Disorder Scale (GAD-7). This scale comprises seven items, with each item response having a four-point Likert scale (from 0 to 3). The total scores for GAD-7 are between 0 and 21, with a higher score indicating a greater severity of anxiety disorders. Anxiety disorders' severity may be classified as minimal (0–4), mild (5–9), moderate (10–14) or severe (15–21) (21). The translated GAD-7 has been pervasively adopted in relation to Chinese children and adolescents, having shown acceptable reliability and validity (20).

#### Covariates

The following variables were treated as covariates in the further analysis, including sex, grade, age, residence, family affluence, as well as living with a parent or not.

### Statistical Analysis

All statistical analysis was performed using SPSS 26.0. Descriptive statistics (percentage for the categorical variable; mean with standard deviation for the continuous variable) were applied to report the sample characteristics. To estimate the associations of sport participation with depressive symptoms and anxiety disorder, the odds ratio (OR) was calculated using Generalized Linear Models with Ordinal Logistic Regression, having controlled all the covariates noted above.

## Results

Data from 1,714 study participants were available for this research. Of the 1,714 study participants, males accounted for 53.4% (see Table 1). Study participants aged 16 were the highest proportion of participants, with adolescents in 10th grades also being the largest proportion. Over 70% of study participants lived in urban areas. 83.8% of study participants resided with their parents. Concerning sports participation, 64.7% of study participants reported that they never engage in sports participation. 52.5% and 64.8% of study participants reported normal severity of depressive symptoms and anxiety disorder, respectively.

**Table 1:** Sample characteristics of this study.

		***n***	**%**
**Gender**
	Boys	915	53.4
	Girls	799	46.6
**Age**
	12 years old	110	6.4
	13 years old	340	19.8
	14 years old	290	16.9
	15 years old	184	10.7
	16 years old	608	35.5
	17 years old	182	10.6
**Grade**
	7th	350	20.4
	8th	415	24.2
	10th	517	30.2
	11th	432	25.2
**Residence**
	Rural	158	9.2
	Suburban	342	20.0
	Urban	1,214	70.8
**Live with parent**
	Yes	1,437	83.8
	No	277	16.2
**Family affluence**
		5.05 ± 1.54	
**Sport participation**
	Never	1,109	64.7
	1–3 times per month	265	15.5
	1–2 times per week	236	13.8
	3 or more times per week	104	6.1
**Depressive symptoms**
	Normal	900	52.5
	Mild	520	30.3
	Moderate	162	9.5
	Moderately severe	62	3.6
	Severe	70	4.1
**Anxiety disorder**
	Normal	1,111	64.8
	Mild	390	22.8
	Moderate	119	6.9
	Severe	94	5.5

**Figure 1:**
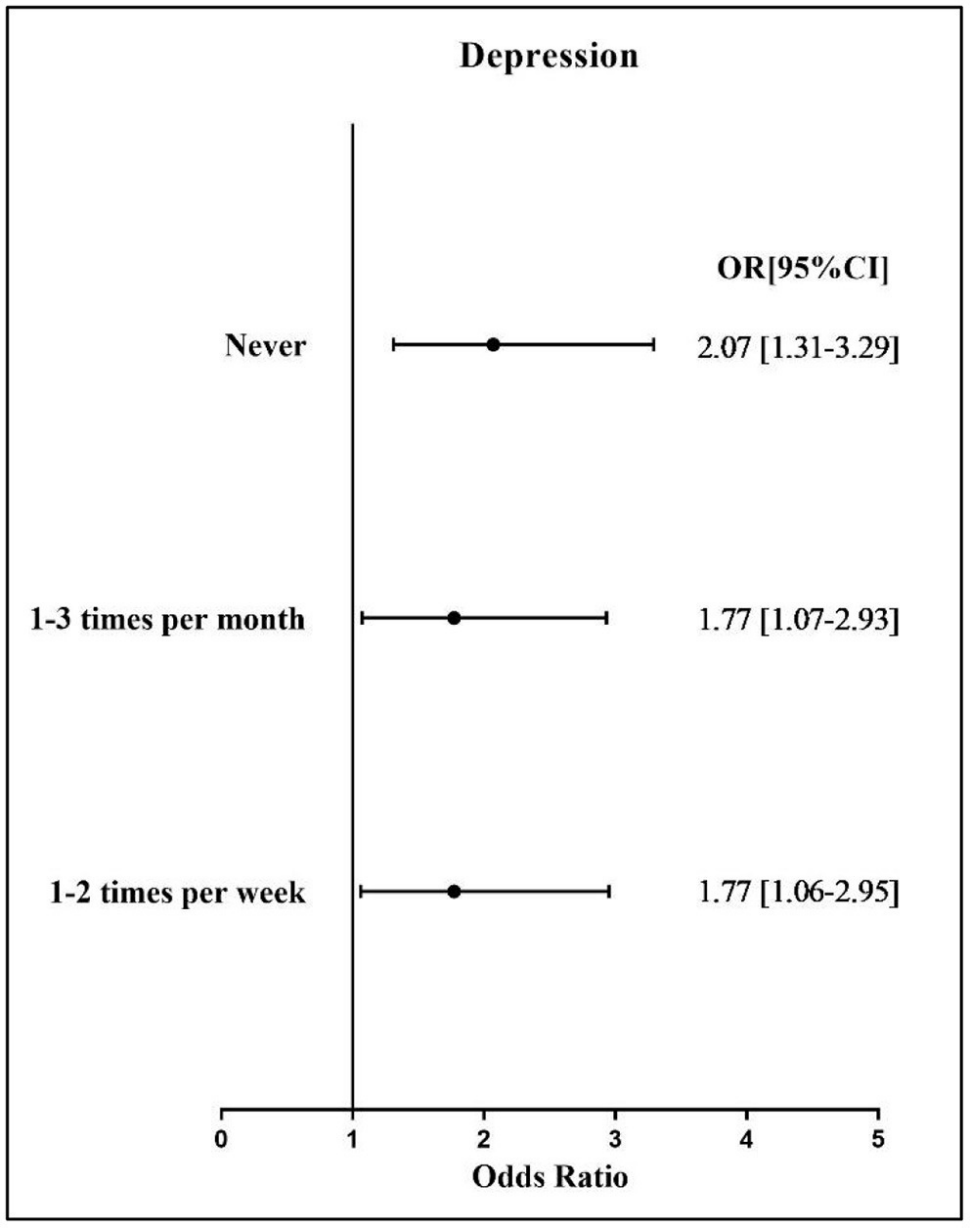
Association between sport participation and depressive symptoms severity. OR, odds ratio; CI, confidence interval. Reference group: 3 or more times per week.

Figure 1 presents the results regarding the correlation between sports participation and depressive symptoms among the study participants. Compared with study participants engaging in sports participation three or more times per week, those with a lower frequency of sport participation had a greater likelihood of reporting higher depressive symptoms (OR for never = 2.07; OR for 1–3 times per month = 1.77; OR for 1–2 times per week = 1.77).

**Figure 2:**
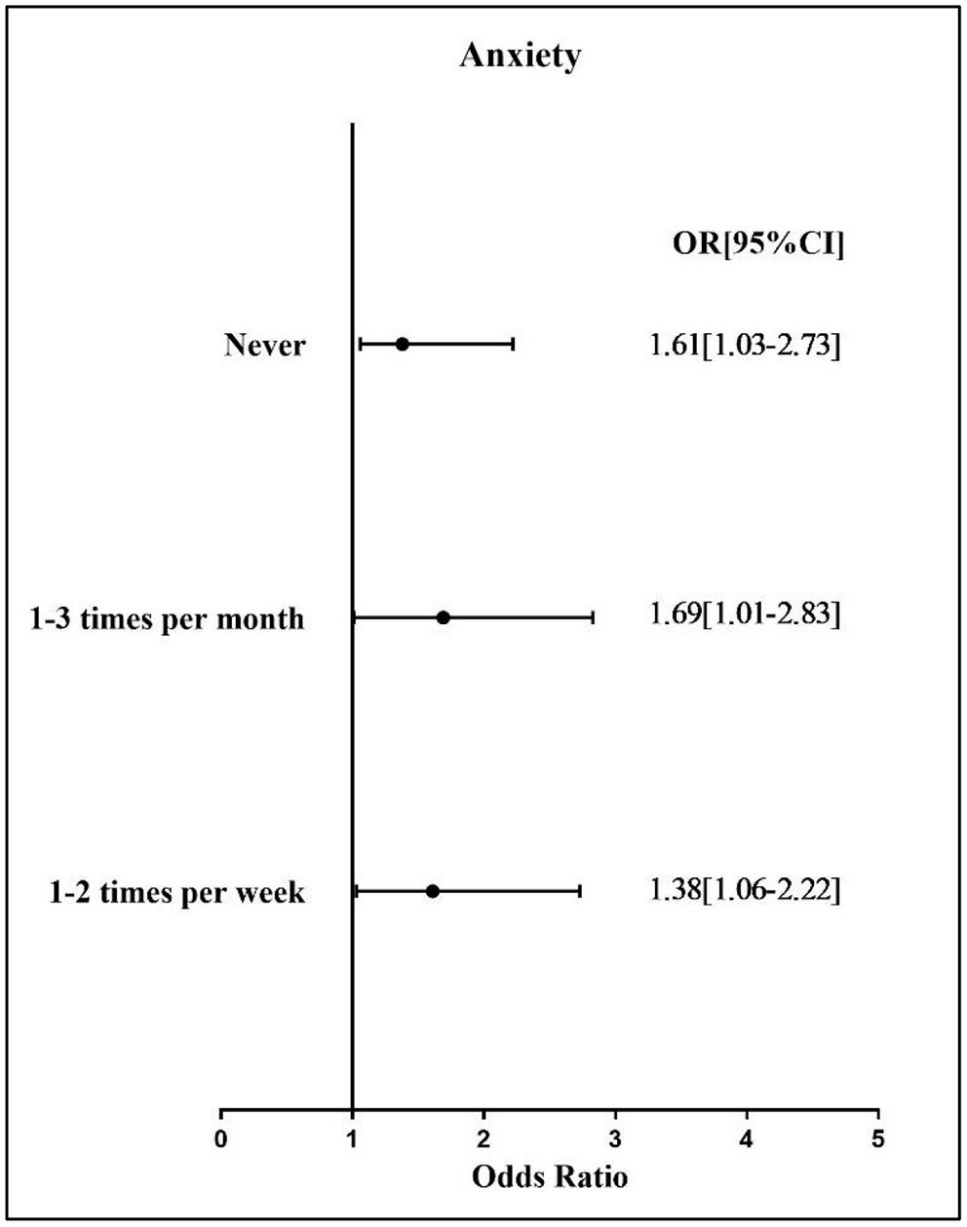
Association between sport participation and anxiety disorder severity. OR, odds ratio; CI, confidence interval. Reference group: 3 or more times per week.

Figure 2 shows the results for the correlation between sports participation and anxiety disorder among the study participants. Compared with those study participants engaging in sports participation three or more times per week, those with a lower frequency of sports participation had a greater likelihood of reporting higher anxiety disorder (OR for never = 1.61; OR for 1–3 times per month = 1.69; OR for 1–2 times per week = 1.38).

## Discussion

This study aimed to analyse the association of sports participation with depressive symptoms and anxiety disorders among adolescents, given that they are at higher risk of depression and anxiety. Our results primarily evidenced that in contrast with no sports participation, participating in more sport-related activities (for example, team or club activities) made the individual less likely to be identified with depressive symptoms. Significantly, we determined that adolescents engaging in greater sports participation (apart from three or more times per week) had lower odds of reporting anxiety disorders, compared with adolescents who never engaged in sports participation. In-depth analysis is presented below.

Our results indicated that sports participation among adolescents could be perceived as a potential means of combatting depressive symptoms. This research finding concerning the negative relationship between sports participation and depressive symptoms established in our research is supported by extant studies (14). For example, a recently published systematic review including meta-analysis revealed a small negative mean correlation between the frequency of sport participation and depression symptoms (ρ = −0.09, 95% CI: −0.11, −0.06) among adolescents (14). A prospective study using cross-lagged analysis identified that greater sports participation predicted subsequent fewer depressive symptoms in adolescents (22). Furthermore, a substantial body of evidence-based cross-sectional studies is consistent with our research findings, thus collectively emphasizing the significance of sports participation for preventing depression (23, 24). Certain potential underpinning mechanisms have been proposed to explain the association between sports participation and depressive symptoms. One possible explanation is that sports participation is enhancing, which partially protect adolescents from depressive symptoms via neurobiological pathways (25). A further potential explanation is that participating in sports-related activities may enable engagement in positive social interactions that reduce individuals' negative emotions, which in turn diminishes depressive symptoms among adolescents (26). However, our study was a cross-sectional survey, which does not allow an exploration of the mechanisms connecting sport participation and depressive symptoms, although answers may be identified from the literature. Notably, in our study we did not observe a dose-dependent association between sport participation and depressive symptoms. Such a dose-dependent association should be expected, because it has been assumed that greater engagement in physical activity has a linear and negative correlation with fewer symptoms of depression, with this assumption being observed in previous studies. Our study identified that the odds of having fewer depressive symptoms when engaging in sport three or more times per week were higher than when participating in sport 1–2 times per week. This indicated that more sport participation may not have a stronger role in preventing depression compared with lower sport participation among adolescents. This significant finding has not been observed in prior studies, because they have suggested that greater sport participation is linked with consecutively decreased odds of depressive symptoms among adolescents. Our study has been unable to explain this interesting finding, therefore it should be addressed in future studies.

Our study results further suggested that sports participation among adolescents is negatively correlated with anxiety disorders. This research finding was consistent with previous studies (27–29). Sports participation may provide opportunities for engagement in physical activity and social interactions that can reduce symptoms of anxiety among adolescents. For example, a recent systematic review suggested that sports participation in adolescents was conversely related to anxiety symptoms (30). Additionally, some single empirical research has offered consistent evidence of this (31, 32).

Regardless of our research findings, certain previous studies have suggested that sports participation leads to detrimental mental health effects due to certain adverse social interactions and experiences (33–35). This point is rational, given that there is much evidence to support it. Studies have proposed that the relationship between sport participation and mental health outcomes among adolescents is affected by the types of sport, the volume of sport, duration of sport, the context of sport and some further factors (for example, measures to improve mental health outcomes, or the operationalisation of sport participation). Therefore, it is suggested that determining the association between sports participation and mental health outcomes is a complex research topic, calling for greater research interests and efforts.

Although the current study identified consistent evidence of the association between sports participation and depressive symptoms and anxiety disorders among adolescents, the research limitations should be mentioned to reach an improved understanding of the research findings. The study limitations include: (1) the cross-sectional design that cannot infer any conclusive evidence; (2) the non-probability sampling method that generated a sample with lower representation; (3) self-reporting measures for collecting data on independent outcomes. To acquire more convincing evidence concerning the association between sports participation and mental health among adolescents, studies with a more optimal design are required that will enable a determination of whether sports participation can be an efficient method for fighting depression and anxiety.

### Practical Implications

In accordance with our research findings, some practical implications may be proposed:

(1) To reduce or prevent depressive symptoms and anxiety among adolescents, encouraging adolescents to participate in more sport-related activities (for example, joining a club or team) is a potentially feasible approach.
(2) Because there are relatively low levels of sport participation among adolescents, efficient intervention should aim at improving sport participation.
(3) Sport participation promotion among adolescents should be integrated into policy design and implementation, to enable young people's overall healthy growth and development.

## Conclusion

Drawing on cross-sectional data for adolescents (high school students) in an economically developed city, this study has provided evidence that an appropriate frequency of engagement in sports may provide a protective measure against depression and anxiety among adolescents, thereby promoting mental health. Although the current study has provided promising evidence, the research findings require replication through enhanced research with an improved study design. Therefore, future researchers are encouraged to design and implement sports participation interventions aimed at promoting mental health among adolescents.

## Data Availability Statement

The datasets presented in this study can be found in online repositories. The names of the repository/repositories and accession number(s) can be found in the article/supplementary material.

## Ethics Statement

The studies involving human participants were reviewed and approved by University Ethics Committee (102772021RT071). Written informed consent to participate in this study was provided by the participants' legal guardian/next of kin. Written informed consent was obtained from the individual(s), and minor(s)' legal guardian/next of kin, for the publication of any potentially identifiable images or data included in this article.

## Author Contributions

GC and LW: conceptualization, writing, investigation, data curation, and resources. LW: methodology, formal analysis, writing—review and editing, and visualization. GC: original draft preparation. Both authors have read and agreed to the published version of the manuscript.

## Conflict of Interest

The authors declare that the research was conducted in the absence of any commercial or financial relationships that could be construed as a potential conflict of interest.

## Publisher's Note

All claims expressed in this article are solely those of the authors and do not necessarily represent those of their affiliated organizations, or those of the publisher, the editors and the reviewers. Any product that may be evaluated in this article, or claim that may be made by its manufacturer, is not guaranteed or endorsed by the publisher.
